# Transfer of Sodium Ion across Interface between Na^+^-Selective Electrode Membrane and Aqueous Electrolyte Solution: Can We Use Nernst Equation If Current Flows through Electrode?

**DOI:** 10.3390/membranes14040074

**Published:** 2024-03-27

**Authors:** Valentina Keresten, Fedor Lazarev, Konstantin Mikhelson

**Affiliations:** Chemistry Institute, St. Petersburg State University, 26 Universiteskij Pr., Stary Peterhof, 198504 St. Petersburg, Russia; v_lukina@list.ru (V.K.); st106290@student.spbu.ru (F.L.)

**Keywords:** Na^+^-selective electrode, ionophore, ion exchanger, interfacial charge transfer, electrochemical impedance, chronopotentiometry

## Abstract

Electrochemical impedance and chronopotentiometric measurements with Na^+^-selective solvent polymeric (PVC) membranes containing a neutral ionophore and a cation exchanger revealed low-frequency resistance, which is ascribed to Na^+^ ion transfer across the interface between the membrane and aqueous solution. The attribution is based on the observed regular dependence of this resistance on the concentration of Na^+^ in solutions. The respective values of the exchange current densities were found to be significantly larger than the currents flowing through ion-selective electrodes (ISEs) during an analysis in non-zero-current mode. This fact suggests that the interfacial electrochemical equilibrium is not violated by the current flow and implies that the Nernst equation can be applied to interpret the data obtained in non-zero-current mode, e.g., constant potential coulometry.

## 1. Introduction

Practical applications of ion-selective electrodes (ISEs) with membranes containing ionophores usually involve recordings of the EMF: a zero-current potentiometric signal (ISE potential) measured against a suitable reference electrode (RE). However, in recent years, ISEs have been increasingly used in non-zero-current modes: voltammetry [1,2,3,4,5,6,7] and chronoamperometry/coulometry [8,9,10,11,12,13,14,15,16,17,18]. A voltammetric analysis with ISEs allows for simultaneous measurements of the concentrations (rigorous activities) of several ions in a sample, using only one electrode [1,2,3,4,5,6]. This is achieved by combining several ionophores, each selective to a particular ion, in the same membrane. The practical advantage of this approach is obvious. The advantage of chronoamperometric/coulometric measurements with ISEs, called constant potential coulometry, is the ability to register very small relative changes in analyte concentrations. A sensitivity of 0.1% was reported for the measurements of K^+^ [11] and Ca^2+^ in blood model solutions [16]. Importantly, voltammetric measurements with ISEs are performed at a scan rate of 100 mV/s, and the currents recorded achieve 1–2 µA [7]. In chronoamperometric measurements, the currents flowing through membranes (and therefore across membrane/solution interfaces) are typically low: 10^−9^–10^−7^ A. However, sometimes they may achieve 10^−4^ A, especially in the reversed amperometric setup [18]. Thus, ISEs may be polarized during measurements whereas the interpretation of the results assumes their Nernstian response to activities of ions in solutions. The Nernstian response, in turn, requires interfacial electrochemical equilibrium between the ISE membrane and the solution. It is therefore necessary to examine the issue of the kinetics of ion transfer across the membrane/solution interface to estimate the values of the exchange current densities and thus to find out how reliable the assumption of a Nernstian response when the current flows through the membrane is.

Sometimes the rate of re-establishment of interfacial electrochemical equilibrium at the interface between the ISE membrane and aqueous solution is estimated from the ISE response time: the time required to achieve a steady EMF value after a change in the solution composition. Furthermore, one can often encounter statements such as the following: fast response times indicate a fast formation of the ion-to-ionophore complex. However, it is widely known that the ISE response time depends on the measurement conditions: whether the sample is stirred, whether the measurements are carried out in a beaker or in a flow-through cell, etc. This dependence suggests that the response time depends primarily on the hydrodynamics of the cell. Therefore, we believe that conclusions about the kinetics of charge transfer across the interface between the ISE membrane and aqueous electrolyte solution in view of the response time are rather dubious. More reliably, these kinetics can be quantified in terms of the exchange current density at the membrane/solution interface [19,20].

In practical measurements with ISEs, a signal is recorded for a time period from a few seconds to a few minutes in each sample or calibration solution, and the average value is considered the analytically relevant signal. Therefore, the kinetics of the interfacial charge transfer does not affect the throughput of the analysis with ISEs. Considered of little practical importance, measurements of the exchange current density at the interface between an ISE membrane and a solution are currently far from mainstream in ISE studies. The data available refer to the results obtained in the 1980s–1990s or in the early 2000s when the mechanism of the ISE response attracted much attention [21,22,23,24,25,26,27,28,29,30,31,32,33,34,35,36,37,38]. These data suggest that the kinetics of the interfacial charge transfer are relatively fast, and the time required to establish the interfacial electrochemical equilibrium lies in the millisecond range, and for sure does not exceed 1 s. However, more studies are needed to address the issue of the interfacial charge transfer at ISE membranes to justify the equilibrium approach to the interpretation of ISE signals obtained in non-zero-current modes.

In this paper, we report on a systematic study of the interfacial properties of Na^+^-selective ISEs. This ISE was chosen as a model system for two reasons. Firstly, the Na^+^ concentration in, e.g., blood serum varies in a relatively narrow range from 120 to 160 mM [39], and thus the variation makes 0.12 logarithmic units only, so chronoampero/coulometric measurements of Na^+^ may be necessary. Secondly, the literature data on the kinetics of charge transfer across the interface between ionophore-based Na^+^-selective membranes and solutions are controversial [33]. Our study was carried out using electrochemical impedance and chronopotentiometric measurements.

Solid-contact construction is predominating in the practical use of ISEs. However, in such ISEs, only the external side of the membrane is in contact with the solution, while the internal side contacts the transducer layer (a conducting polymer or a carbonaceous material or else). The current flows through the whole electrode, and therefore the measured signal depends on the processes at both sides of the membrane. Therefore, it is hardly possible to extract data referring to the membrane/solution interface from signals recorded with solid-contact ISEs. So-called conventional ISEs, i.e., with an internal aqueous solution and an internal reference electrode, are more suitable for such studies. Furthermore, for a correct interpretation of the results, the compositions of the internal and the external solutions must be the same so that the two interfaces are identical. Therefore, this study was performed with conventional ISEs.

Here, for the first time, we report on low-frequency resistances, which regularly depend on the concentration of Na^+^ ions in aqueous solutions. We ascribe these resistances to the interfacial charge transfer, and using these results, we estimate the exchange current densities at the interface between Na^+^-selective membranes and aqueous solutions.

## 2. Materials and Methods

The sodium ionophore X: 4-tert-Butylcalix[4]arene-tetraacetic acid tetraethyl ester (Na X), potassium tetrakis-*p*-Cl-phenylborate (KClTPB), lipophilic salt tetradodecylammonium tetrakis-*p*-Cl-phenylborate (ETH 500), solvent plasticizer 2-nitrophenyloctyl ether (oNPOE), and high-molecular-weight poly(vinyl chloride) (PVC) were Selectophore-grade reagents from Merck (Darmstadt, Germany). The volatile solvents were extra-pure cyclohexanone (CH) and HPLC-grade tetrahydrofuran (THF) from Vekton (St. Petersburg, Russia), freshly distilled before use. The inorganic salts were from Reaktiv (Moscow, Russia). The MgCl_2_∙6 H_2_O was from AppliChem (Darmstadt, Germany) and titrated with EDTA to determine the exact content of MgCl_2_ in the chemical. All the aqueous solutions were prepared with deionized water with a resistivity of 18.2 MΩ∙cm (Milli-Q Reference, Millipore, France).

The membrane cocktails were prepared by dissolving appropriate amounts of PVC and oNPOE in THF. After that, Na X, KClTPB, and ETH 500 were added as appropriate aliquots of stock solutions in cyclohexanone. In this way, weighing small amounts was avoided, ensuring high accuracy of the membrane composition. To obtain the membranes, the cocktails were stirred for 30 min using roller-mixer Selecta Movil Rod (Barcelona, Spain) and then poured into 30 mm diameter Teflon Petri dishes. The dishes were covered with filter paper to slow down the evaporation of THF. The complete evaporation of THF and CH took 2 days, and after that, master membranes with a thickness of about 70 μm were obtained. Four membranes were prepared: membrane M1 contained Na X: a Na^+^-selective ionophore, KClTPB: a cation exchanger (ionic sites), and ETH 500: a lipophilic electrolyte. Membrane M2 was similar to M1 but did not contain ETH 500, M3 contained only a cation exchanger, and M4 contained only an ionophore. The membrane compositions are specified in Table 1.

The electrodes were prepared by cutting disks with a diameter of 10 mm from the master membrane and gluing them to PVC bodies with an outer diameter of 12 mm and an inner diameter of 8 mm. A solution of PVC in CH was used as the glue. The electrodes were filled with 0.01 M NaCl. A chlorinated silver wire in a polypropylene body was used as the internal reference electrode.

Zero-current potentiometric measurements were performed with the Lawson EMF-16 16-channel potentiometric station (Lawson Labs, Malvern, PA, USA). The reference electrode was a single junction Ag/AgCl electrode in 3.5 M KCl with a salt bridge with a limited leak of KCl. Potentiometric calibrations in pure NaCl solutions were performed from 10^−1^ M down to 10^−8^ M NaCl using an automatic burette Metrohm 700 Dosino controlled by the Metrohm 711 Liquino Controller. Once the value 10^−8^ M NaCl was achieved, a suitable aliquot of MgCl_2_ was added to the solution. This was conducted to estimate the possible interference from Mg^2+^ with the ISE response to Na^+^ ions. The selectivity of Na^+^ over K^+^ was measured in the same way.

Chronopotentiometric and electrochemical impedance studies were performed with Potentiostat-Galvanostat Autolab 302N with a frequency response analyzer module FRA 2 (Metrohm, Herisau, Switzerland). The chronopotentiometric and impedance measurements were carried out using an ordinary three-electrode cell. The compositions of the internal solution (inside the ISE) and the external solution (in the beaker) were the same, so the ISE membranes were in contact with identical solutions from both sides. In these measurements, the internal and the external reference electrodes were also identical: chlorinated silver wires. The counter electrode was a glassy carbon rod. The concentration of NaCl in the chronopotentiometric and impedance measurements varied from 10^−1^ to 10^−6^ M. At chloride concentrations below 10^−3^ M, especially in non-zero-current measurements, Ag/AgCl electrodes may be polarized. To prevent this effect, NaCl solutions used in chronopotentiometric and impedance measurements always contained at least 2 mM Cl^−^ added as 10^−3^ M MgCl_2_.

The impedance measurements were made in potentiostatic mode with an excitation magnitude of ±5 mV around the open-circuit potential (OCP), over the frequency range from 10 kHz to 0.01 Hz. Regarding the chronopotentiometric measurements, the OCPs were recorded for the first 3 s, then the current was turned on, and the potential was registered for 10 s. After that, the current was turned off, and the potential was registered for another 10 s. The time resolution was 0.005 s.

Before the studies, the ISEs were soaked in 0.01 M NaCl for 3 days. In between the measurements, the ISEs were stored in the same solution. All the measurements were carried out in plastic beakers with a volume of 50 mL at room temperature (22 ± 1 °C). Three replicate electrodes with each membrane composition were used in this work.

The activity coefficients of the Na^+^ ions in the solutions were calculated according to the 3rd approximation of the Debye–Hückel theory):

(1) $$log\left(\gamma_{Na}\right)=-\frac{0.512\cdot \sqrt{I}}{1+0.328{\cdot a}_{Kiell}^{Na}\cdot \sqrt{I}}+0.05\cdot I$$

Here, $\gamma_{Na}$ is the ion activity coefficient, $z_{I}$ is the ion charge number, I is the solution ionic strength, and $a_{Kiell}^{Na}$ is the Kielland parameter for Na^+^ [40,41].

## 3. Results and Discussion

### 3.1. Potentiometric Studies

**Figure 1:**
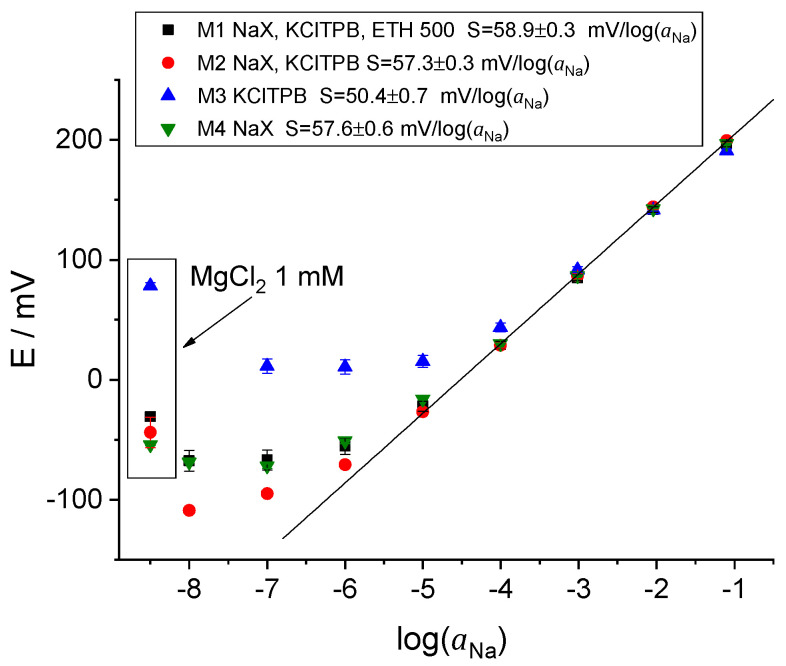
Potentiometric response of freshly prepared ISEs with membranes M1, M2, M3, and M4. Data inside the rectangle refer to EMF values recorded in pure 1 mM MgCl_2_.

Freshly prepared ISEs were calibrated potentiometrically in pure NaCl solutions. The example of the time traces of the EMF vs. time along with the calibration of the ISE with membrane M2 in NaCl solutions is shown in Figure S1, Supplementary Material. The calibration plots are presented in Figure 1.

Not surprisingly, the ISEs with the membrane M1 containing the ionophore, the ion exchanger, and the lipophilic electrolyte ETH 500, as well as M2 without ETH 500, showed a near-Nernstian response over the NaCl concentration range from −5 to −1 (in logarithmic units) and a good selectivity (measured by the separate solutions method [42]) to Na^+^ over K^+^ and Na^+^ over Mg^2+^: $\log\left(K_{Na/K}\right)\approx -2.3$, $\log\left(K_{Na/Mg}\right)\approx -4$. The obtained values are consistent with the literature data [43]. The potentiometric response of the ISEs with membranes M1 and M2 was close to Nernstian during more than two months, whereas the ISEs with membrane M3 (only KClTPB) showed a sub-Nernstian slope, see Figure S2A–C, Supplementary Material. At the same time, the bulk resistance of membranes M1 and M2 increased over time, as discussed in Section 3.2 and Section 3.3.

Interestingly, the ISEs with the membrane M4 (only the ionophore) showed a good cationic slope, as shown in Figure S2D (Supplementary Material), and were selectivity similar to those with membranes M1 and M2. The membranes based on neutral ionophores are usually doped with ion exchangers to ensure the Donnan exclusion of co-ions (Cl^−^ in this case). Normally, this is considered necessary to obtain the Nernstian response of an ISE. However, in the early days of ionophore-based ISEs, the membranes did not contain intentionally added ion exchangers [44,45]. A near-Nernstian response was explained as follows: water in water droplets inside the membrane dissociates into H^+^ and OH^−^, and cations from the solution partially replace the H^+^ ions in the membrane, whereas the OH^−^ ions are entrapped in water droplets, effectively acting as quasi-ion exchangers [46]. Later it was shown that the response of the membranes without ion exchangers is due to the inevitable presence of impurities [47,48,49]. The concentration of the impurities in the PVC membranes plasticized with oNPOE is roughly 0.16 mM; in membranes with bis(2-ethylhexyl)sebacate and with bis(2-ethylhexyl)phthalate, it is roughly 0.01–0.06 mM [49]. Obviously, membranes with such a low content of charged species should have a high bulk resistance. This expectation turned out to be true, as described in Section 3.2.

### 3.2. Electrochemical Impedance Studies

**Figure 2:**
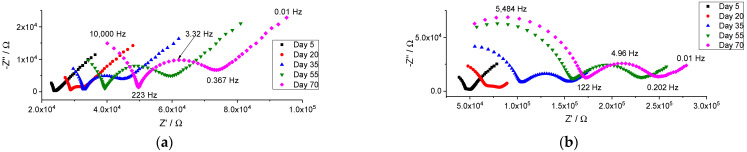
Change in the impedance spectra of the electrodes during the period of 70 days, registered in 0.01 M NaCl. (**a**) Membrane M1; (**b**) membrane M2.

Although the potentiometric response of ISEs with membranes M1 and M2 was stable and close to Nernstian during the whole period of studies, the resistance of the membranes increased significantly over time. The impedance spectra of freshly prepared ISEs contained semicircles at high frequencies, but it is difficult to interpret the low-frequency part of the spectra (Figure S3, Supplementary Material). Over time, not only did the bulk resistance of the ISEs increase, but well-developed semicircles also appeared at low frequencies, as shown in Figure 2.

**Figure 3:**
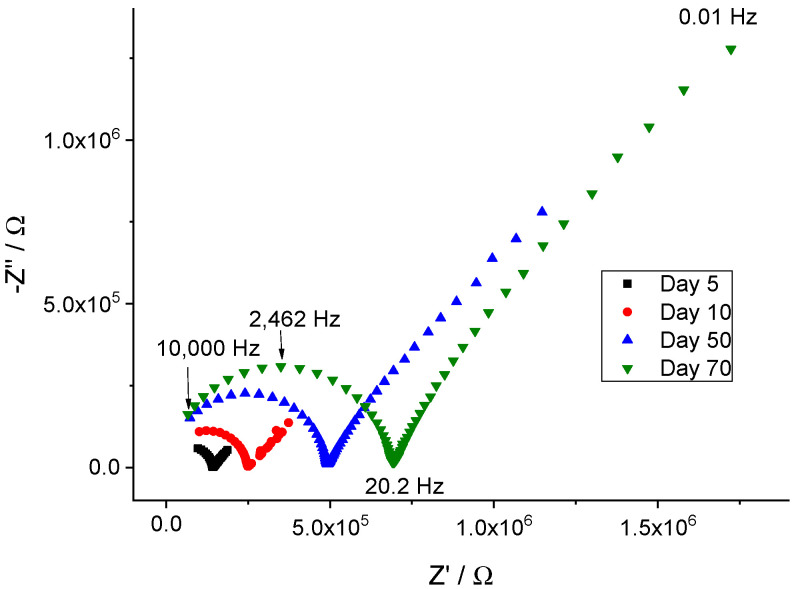
Change in the impedance spectra of the electrodes with membrane M3 during the period of 70 days, registered in 0.01 M NaCl.

The same trend, an increase in membrane resistance over time, was observed for membrane M3 containing only an ion exchanger, see Figure 3. Unlike the ISEs with membranes M1 and M2, electrodes with membrane M3 did not show a well-developed low-frequency semicircle. In the cases of M1 and M2, the low-frequency semicircle appears in frequency ranges from 200 to 0.4 Hz (M1) or from 100 to 0.2 Hz (M2). In the case of M3, the high-frequency semicircle is larger and refers to frequencies down to 20 Hz. Apparently, the time constants of the two semicircles for membrane M3 do not differ enough, and therefore, the low-frequency part of the spectra of the ISE with membrane M3 is hidden within the high-frequency part.

Apparently, the main reason for the increase in the membrane bulk resistance over time is the leakage of ion-exchanger sites from membranes, due to the insufficient lipophilicity of ClTPB^−^ [50,51]. Estimations made using Molinspiration Cheminformatics [52] give the lipophilicity values (logP) of 10.13 for the Na X ionophore, 6.64 for KClTPB, and 5.36 for oNPOE. Thus, one can expect that the ionic sites and the plasticizer tend to leak from the membrane to a much greater extent than the ionophore. The leakage of the ion-exchanger sites directly results in an increase in the membrane resistance due to a decrease in the concentration of charge carriers. The leakage of the plasticizer causes the increase in the resistance via the increased viscosity of the membrane. Bearing in mind the relatively small amount of ion exchanger in membranes, its leakage appears to be the main reason for the gradual increase in the membrane resistance.

**Figure 4:**
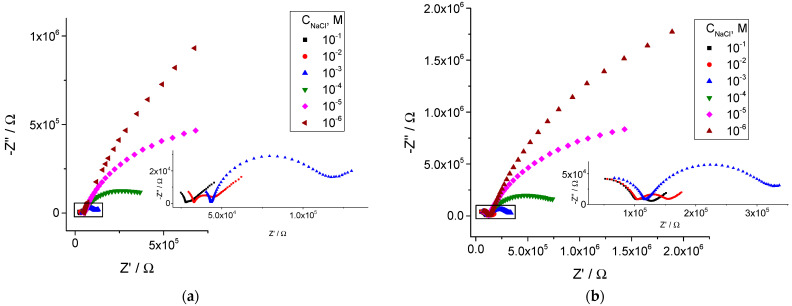
Impedance spectra of the electrodes in solutions with different concentrations of NaCl with constant background of 0.001 M MgCl_2_, day 35. The insets show the impedance spectra recorded at NaCl concentrations 0.1–0.001 M on a larger scale. (**a**) Membrane M1; (**b**) membrane M2.

The increase in the bulk resistance, besides the leakage of the ion exchanger, can be due to the gradual uptake of water from solutions. We have shown earlier that water uptake results in an increase in the bulk resistance of the K^+^, Ca^2+^, Cd^2+^, and NO_3_^−^ selective membranes [38,53]. Importantly, water is non-uniformly distributed within ISE membranes: layers in the vicinity of the membrane/solution interface are enriched with water [54,55,56]. This may be the reason for changes in the ion transfer across the membrane/solution interface, in addition to the gradual increase in the membrane bulk resistance. This results in the development of low-frequency semicircles, which can be seen within the frequency range from 200 to 1 Hz. Nyquist plots of the impedance of the ISE with membranes M1 and M2 registered at different concentrations of NaCl on the 35th day (the middle of the overall period of studies) are presented in Figure 4.

**Figure 5:**
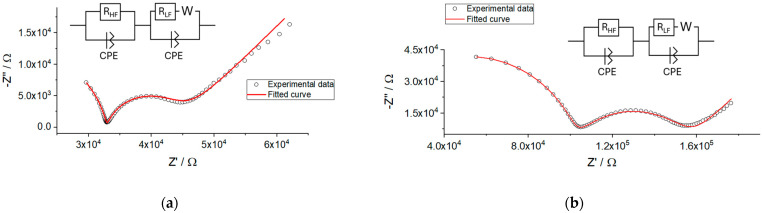
Experimental (black dots) and fitted (red curve) impedance spectra recorded in 0.01 M NaCl with 0.001 M MgCl_2_, day 35. (**a**) Membrane M1; (**b**) membrane M2.

Examples of the impedance spectra of the ISEs with membranes M1 and M2, together with the circuit used for fitting, are shown in Figure 5. One can see that the circuit containing a high-frequency resistance (R_HF_) in parallel with a high-frequency constant phase element (CPE_HF_), as well as analogous low-frequency components (R_LF_, CPE_LF_) and Warburg resistance, allows for a good fitting of the experimental results. Factor n of CPE_HF_ was about 0.9, close to an ideal capacitor. The respective values were roughly 10^−10^ F. We therefore ascribe R_HF_ to the bulk resistance of the membrane and CPE_HF_ to its geometric capacitance. Factor n of CPE_LF_ was about 0.6–0.8, and the values of CPE_LF_ varied from 10^−8^ to 10^−7^ F. Obviously, CPE_LF_ cannot be treated as an ideal capacitor, but its value anyway allows one to ascribe this element to the capacitance of the double electric layer at the membrane/solution interface. This, together with a strong dependence of R_LF_ on the concentration of NaCl, assumes that the low-frequency part of the spectra refers to the interface between the membrane and solution.

**Figure 6:**
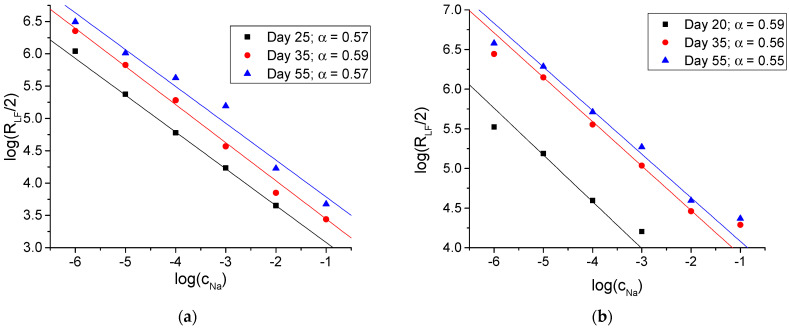
The dependence of low-frequency resistance on the concentration of NaCl, registered on different days during the period of this study. (**a**) Membrane M1; (**b**) membrane M2.

If indeed R_LF_ refers to the transfer of Na^+^ ions across the interface between the membrane and aqueous solution, its value must regularly depend on the concentration of Na^+^ ions. The symmetry of the measurement setup (the membrane is in contact with two identical solutions–internal and external) implies the following relation between the charge transfer resistance (R_CT_) and R_LF_: R_CT_ = R_LF_/2. The values of R_LF_ plotted vs. the concentration of NaCl (log–log) are shown in Figure 6.

One can see that the values of the low-frequency impedance gradually increase during the whole period of studies. However, $R_{LF}$, the low-frequency resistance, always showed a regular dependence on $C_{Na}$, the concentration of Na^+^ ions in solutions (the solid lines in Figure 6a,b), and complies with Equation (2):

(2) $$log\left(R_{LF}/2\right)=log\left(R_{LF}^{0}/2\right)+\alpha log(C_{Na})$$

The regular dependence of the low-frequency resistance ($R_{LF}$) on the concentration of Na^+^ ions in solutions (see Figure 6) is in line with the Butler–Volmer theory [57]. This confirms ascribing this resistance to the interfacial charge transfer. The slope $\alpha =d\left(logR_{LF}\right)/d\left(logC_{Na}\right)$ of about −0.6 suggests a fairly symmetric activation barrier of the interfacial transfer of Na^+^ ions from the solution to the membrane and back. Over time, both the bulk and interfacial resistances increase so that clear low-frequency semicircles appeared also at 0.01 and 0.1 M NaCl, as can be seen in Figure 2. The dependence of the low-frequency resistance on the Na^+^ concentration (log–log) remains linear with the same slope, although the very values of $R_{LF}$ during one month increased in one order of magnitude, see Figure 6a,b.

We used the values of the low-frequency resistance to calculate the standard values of the exchange current density using Equation (3) [57]:

(3) $$i_{Na,0}^{0}=RT/\left(F\cdot \frac{1}{2}R_{LF}^{0}\cdot A\right),$$

Here *R*, *T*, and *F* are the gas constant, temperature, and the Faraday constant, respectively, and *A* is the electrode surface area (2.6 cm^2^). The values of $R_{LF}^{0}$, the low-frequency resistance extrapolated to 1 M NaCl, and those of the standard exchange current density are presented in Table 2.

Thus, the standard values of the exchange current densities on membranes aged 1 month or less exceed the currents, which flow during chronoamperometric measurements in two or three orders of magnitude, and those in voltammetric measurements in one order of magnitude. This result is in favor of using the Nernst equation when interpreting ISE signals measured in non-zero-current mode. However, this may not be the case with aging electrodes, so the lifetime of the ISE in the non-zero-current mode may be shorter than in the traditional potentiometric mode. On the other hand, the use of ionic sites with lipophilicities higher than KClTPB, e.g., sodium tetrakis[3,5-bis-(trifluoromethyl)phenyl]borate, may allow for a large improvement in the ISE lifetime.

### 3.3. Chronopotentiometric Studies

**Figure 7:**
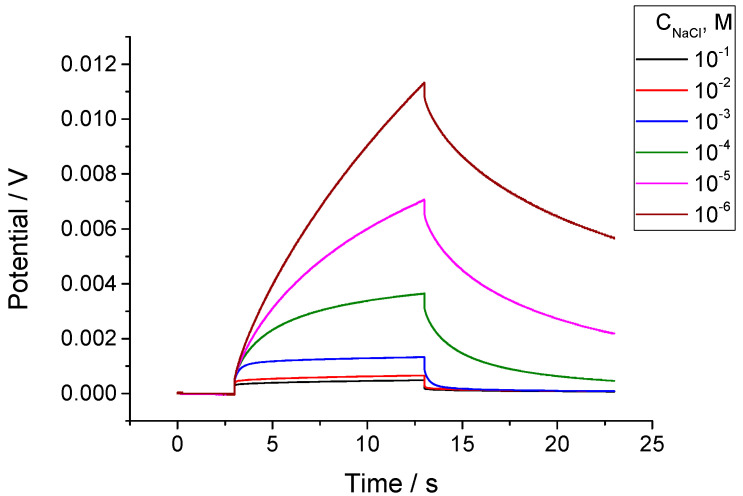
Chronopotentiometric curves recorded with ISE M1 in NaCl solutions with concentrations from 10^−1^ M to 10^−6^ M, with constant background of 1 mM MgCl_2_. Polarizing current 10^−8^ A (current density 10^−8^ A/cm^2^), time resolution 0.005 s.

Chronopotentiometric measurements carried out with ISEs with the membrane M1 revealed the same trend as obtained by impedance studies: drastic changes in the magnitude of polarization and relaxation along with the change in the NaCl concentration in solutions, see Figure 7. By analogy with the impedance measurements, the chronopotentiometric data also showed an increase in the resistance along with the time of the ISEs’ contact with the solution, as shown in Figure S4, Supplementary Material.

The polarization and relaxation parts of the curves including the respective Ohmic drops were fitted to the equation below, which combines the electrochemical impact (decaying exponent) and concentration polarization, which evolves with the square root of time:

(4) $$E=i\cdot R_{Ohm}+i\cdot \left(R_{expon}\left(1-exp\left(-t/\tau \right)\right)+N\surd t\right)$$

Here, *E* is the potential; *i* is the polarizing current density; $R_{Ohm}$ is the resistance obtained from the Ohmic drop; $R_{expon}$ is the resistance, which refers to the decaying exponent with *τ*—the characteristic time; and *t* is the time from the moment when the current was turned on or off. Factor *N* is dependent on *A*—the electrode cross-section area, *C_I_* is the concentration of the ionic species in the membrane, and *D_I_* is their diffusion coefficient: $N=2\pi^{1/2}\frac{F}{RT}{\left(AC_{i}\sqrt{D_{i}}\right)}^{-1}$ [57]. Examples of the experimental and fitted curves are shown in Figure S5, Supplementary Material.

**Figure 8:**
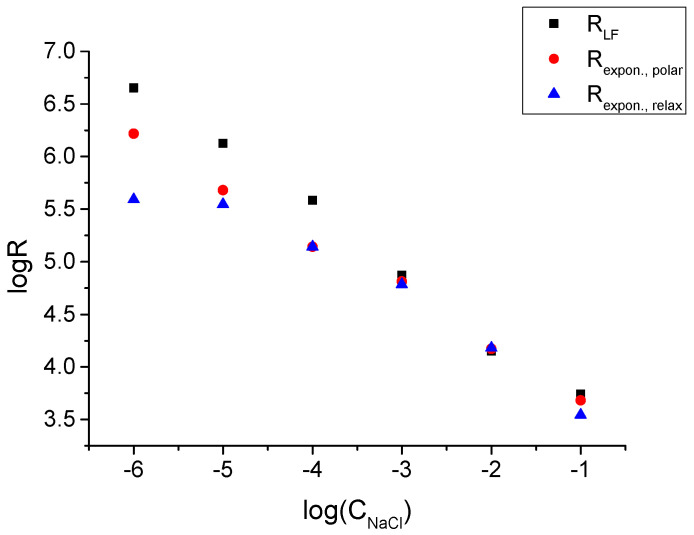
Comparison of $R_{LF}$ low-frequency resistance obtained from impedance measurements with $R_{expon}$ values obtained by fitting polarization and relaxation parts of chronopotentiometric curves. Data refer to membrane M1, day 35.

The nature of the exponential part of the polarization and relaxation curves must be the same as that of the low-frequency resistance in the impedance: Na^+^ ion transfer across the membrane/solution interface. The data shown in Figure 8 confirm this expectation: the values of $R_{expon}$ show the same trend as $R_{LF}$—linear dependence (log–log) on the concentration of NaCl. The respective values are also relatively close to each other except $R_{expon}$ obtained from the relaxation curve at $C_{NaCl}=1\cdot 10^{-6}$ M.

## 4. Conclusions

The study of Na^+^-selective membranes performed by electrochemical impedance spectroscopy and chronopotentiometry allowed us to obtain data on the interface between the membrane and aqueous solution. A low-frequency resistance ($R_{LF}$) at the interface of the sodium-selective ISEs can be clearly seen in the electrochemical impedance, especially at Na^+^ ion concentrations below 0.01 M, or on relatively “aged” ISEs after 3 weeks in continuous contact with the solution. The same is true regarding $R_{expon}$: the resistance obtained from the exponentially decaying part of the polarization and relaxation curves in the chronopotentiometric measurements. These resistances are dependent on the Na^+^ concentration in solutions so that the logarithm of the resistance is linearly dependent on the logarithm of the solution concentration. This allows one to ascribe the values of the low-frequency resistance measured by impedance: $R_{LF}$ and $R_{expon}\;$obtained by chronopotentiometric measurements to the process of Na^+^ ion transfer across the membrane/solution interface.

Values of the exchange current density estimated from $R_{LF}$ vary within the range from ≈60 µA/cm^2^ for ISEs aged 3 weeks to ≈3 µA/cm^2^ for ISEs aged 2 months. Apparently, this change, as well as the increase in the high-frequency resistance that we ascribe to the bulk of the membrane, is caused by the leakage of ionic sites due to the insufficient lipophilicity of KClTPB. Therefore, the replacement of KClTPB with more lipophilic ionic sites like, e.g., sodium tetrakis[3,5-bis-(trifluoromethyl)phenyl]borate appears very promising in view of maintaining a high exchange current over longer periods of time. Anyway, even for membranes with KClTPB, the estimated values of the exchange current densities significantly exceed the current densities used during analysis with ISEs by the constant potential coulometry method. Also, frequencies characteristic of low-frequency semicircles (ca. 200–1 Hz) imply a short equilibration time at the membrane/solution interface. These facts indicate electrochemical equilibrium at the interface between the ISE and solution and justify the use of the Nernst equation in the interpretation of ISE signals recorded in non-zero-current measurements, e.g., with the constant potential coulometry method.

Tentatively, one can conclude that the facilitation of the interfacial ion transfer (in the kinetic sense) is due to the ion exchanger rather than to the ionophore. This, however, requires further studies with variations in the concentration of the electrolyte in the solution together with variations in the concentrations of the ionophore and ion exchanger in the membrane.

## Figures and Tables

**Table 1:** Membrane compositions.

Membrane	Na X	KClTPB	ETH 500	oNPOE	PVC
M1	15 mmol 1.0%	7.5 mmol 0.2%	7.5 mmol 0.6%	65.5%	32.7%
M2	15 mmol 1.0%	7.5 mmol 0.2%	-	65.9%	32.9%
M3	-	7.5 mmol 0.2%	-	66.5%	33.3%
M4	15 mmol 1.0%	-	-	66.0%	33.0%

**Table 2:** Low-frequency resistance ($R_{LF}^{0}/2$), transfer coefficient (α), and the standard exchange current density ($i_{Na,0}^{0}$) at the interfaces between membranes M1 and M2 and NaCl solution, measured on different days during the study.

Membrane, Day	$log\left(R_{LF}^{0}/2\right)$	α	$R_{LF}^{0}/2$, kΩ	$i_{Na,0}^{0}$, µA/cm^2^
M1, day 25	2.51	−0.57	0.324	30.0
M1, day 35	2.86	−0.59	0.724	13.6
M1, day 55	3.22	−0.57	1.66	5.95
M2, day 20	2.22	−0.59	0.166	59.5
M2, day 35	3.35	−0.56	2.24	4.41
M2, day 55	3.53	−0.55	3.39	2.91
M2, day 70	3.51	−0.56	3.24	3.05

## Data Availability

The original contributions presented in the study are included in the article/Supplementary Material, further inquiries can be directed to the corresponding author/s.

## Supplementary Materials

The following supporting information can be downloaded at https://www.mdpi.com/article/10.3390/membranes14040074/s1: Figure S1. Calibration of ISE with membrane M2: time traces; Figure S2: Potentiometric response of ISEs with membranes M1 (A), M2 (B), M3 (C), and M4 (D) at different times during the study; Figure S3: Impedance spectra of ISEs after 5 days in contact with 0.01 M NaCl. Measurements in 0.01 M NaCl with 1 mM MgCl_2_, inset shows enlarged spectra of ISEs with membranes M1 and M2; Figure S4: Chronopotentiometric curves recorded for ISE with membrane M1 at different days during the study, polarizing current 10^−8^ A, 0.01 M NaCl with 1 mM MgCl_2_; Figure S5: Examples of fitting chronopotentiometric curves to Equation (4). ISE with membrane M1 NaCl solutions with 1 mM MgCl_2_, 0.1 M NaCl (A), and 1 µM NaCl (B).
