# Supplementary material for: Transfer of Sodium Ion across Interface between Na+-Selective Electrode Membrane and Aqueous Electrolyte Solution: Can We Use Nernst Equation If Current Flows through Electrode?
Source: Membranes (Basel). 2024 Mar 27;14(4):74. doi: 10.3390/membranes14040074 (PMC11052482; doi:10.3390/membranes14040074)
Supplement: Supplementary file 1 [file membranes-14-00074-s001.zip › membranes-2899250-supplementary.pdf]

# Transfer of Sodium Ion across Interface between $Na^+$-Selective Electrode Membrane and Aqueous Electrolyte Solution: Can We Use Nernst Equation If Current Flows through Electrode?

**Valentina Keresten, Fedor Lazarev and Konstantin Mikhelson ***

Chemistry Institute, St. Petersburg State University, 26 Universiteskij Pr., Stary Peterhof, 198504 St. Petersburg, Russia; v_lukina@list.ru (V.K.); st106290@student.spbu.ru (F.L.)

* Correspondence: konstantin_mikhelson@outlook.com or k.mikhelson@spbu.ru; Tel.: +7-921-757-36-32

## Supplementary Material

Content

Figure S1. Calibration of ISE with membrane M2: time traces.

Figure S2. Potentiometric response of ISEs with membranes M1 (A), M2 (B), M3 (C) and M4 (D) on different times during the study.

Figure S3. Impedance spectra of ISEs after 5 days in contact with 0.01 M NaCl. Measurements in 0.01 M NaCl with 1 mM $MgCl_2$, inset shows enlarged spectra of ISEs with membranes M1, M2.

Figure S4. Chronopotentiometric curves recorded for ISE with membrane M1 at different days during the study, polarizing current $10^{-8}$ A, 0.01 M NaCl with 1 mM $MgCl_2$.

Figure S5. Examples of fitting chronopotentiometric curves to Eq. 4 (see main text). ISE with membrane M1 NaCl solutions with 1 mM $MgCl_2$, 0.1 M NaCl (A) and 1 µM NaCl (B).

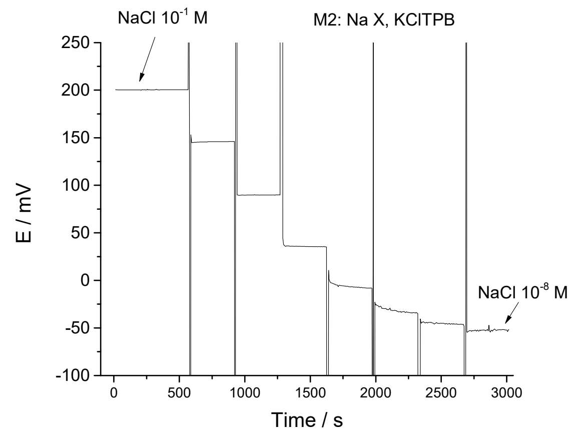


**Figure S1.** Calibration of ISE with membrane M2: time traces.

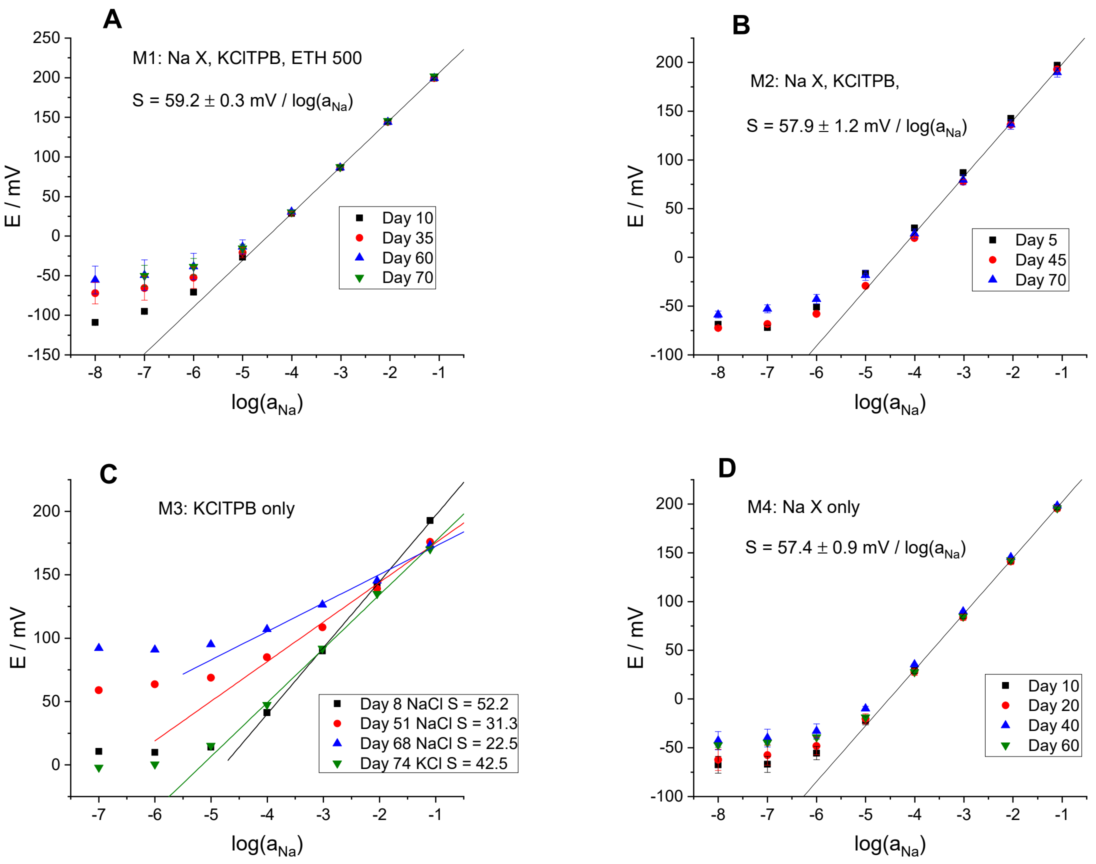


**Figure S2.** Potentiometric response of ISEs with membranes M1 (A), M2 (B), M3 (C) and M4 (D) on different times during the study.

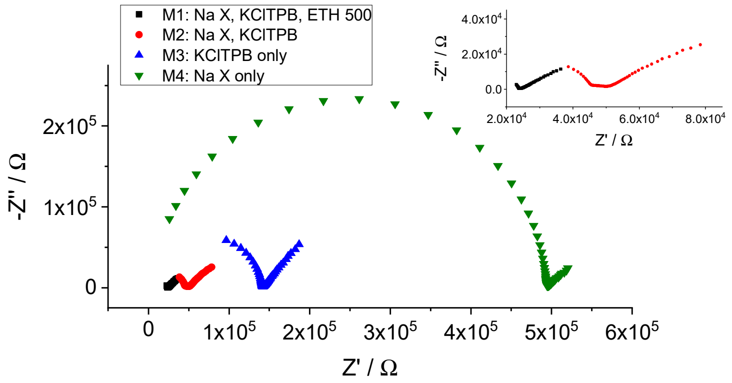


**Figure S3.** Impedance spectra of ISEs after 5 days in contact with 0.01 M NaCl. Measurements in 0.01 M NaCl with 1 mM $MgCl_2$, inset shows enlarged spectra of ISEs with membranes M1, M2.

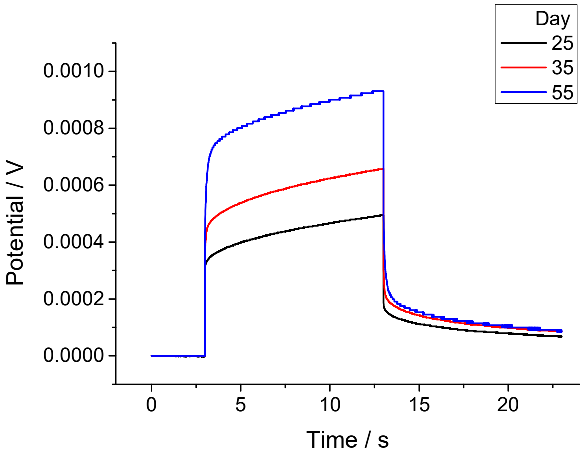


**Figure S4.** Chronopotentiometric curves recorded for ISE with membrane M1 at different days during the study, polarizing current $10^{-8}$ A, 0.01 M NaCl with 1 mM $MgCl_2$.

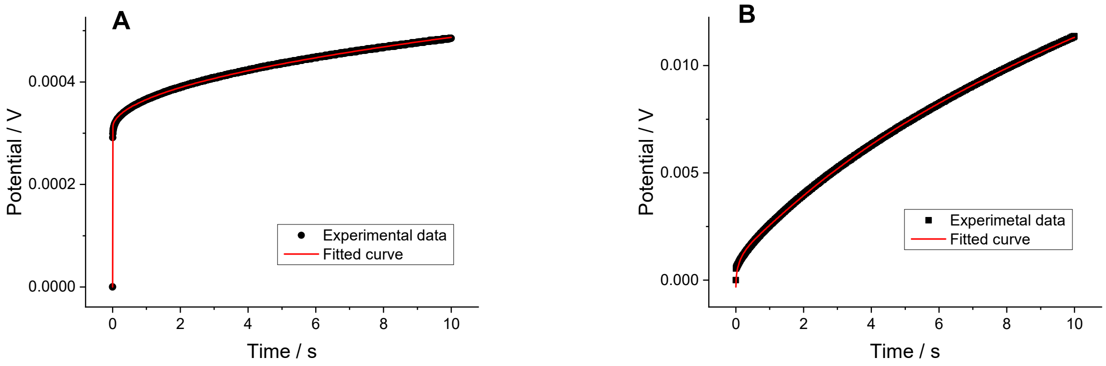


**Figure S5.** Examples of fitting chronopotentiometric curves to Eq. 4 (see main text). ISE with membrane M1 NaCl solutions with 1 mM $MgCl_2$, 0.1 M NaCl (A) and 1 µM NaCl (B).
